# The immune system in cancer metastasis: friend or foe?

**DOI:** 10.1186/s40425-017-0283-9

**Published:** 2017-10-17

**Authors:** Louise M.E. Janssen, Emma E. Ramsay, Craig D. Logsdon, Willem W. Overwijk

**Affiliations:** 1 Departments of Melanoma Medical Oncology, The University of Texas M.D. Anderson Cancer Center, Houston, TX USA; 2 Cancer Biology, The University of Texas M.D. Anderson Cancer Center, Houston, TX USA; 3 The University of Texas MD Anderson Cancer Center UTHealth Graduate School of Biomedical Sciences, Houston, TX USA

## Abstract

Metastatic disease is the leading cause of death among cancer patients and involves a complex and inefficient process. Every step of the metastatic process can be rate limiting and is influenced by non-malignant host cells interacting with the tumor cell. Over a century ago, experiments first indicated a link between the immune system and metastasis. This phenomenon, called concomitant immunity, indicates that the primary tumor induces an immune response, which may not be sufficient to destroy the primary tumor, but prevents the growth of a secondary tumor or metastases. Since that time, many different immune cells have been shown to play a role in both inhibiting and promoting metastatic disease. Here we review classic and new observations, describing the links between the immune system and metastasis that inform the development of cancer therapies.

## Background

### Future and past: A link between the immune system and metastasis

One of the biggest obstacles to finding a cure for most solid cancers is not the removal of the primary tumor, but the elimination of metastases [1]. If tumors were non-metastatic, complete surgical removal would often lead to complete cure. Therefore, understanding and controlling metastatic disease is essential for clinical practice. Metastases arise from solitary solid tumors when cancer cells undergo distinct changes and progress through a multi-step metastatic cascade, creating disseminated tumors that are difficult to treat. The metastatic process consists of 1) invasion of metastatic cancer cells into the local tissue at the primary tumor site, 2) intravasation of metastatic cancer cells into blood or lymph vessels, 3) survival in the circulation, 4) extravasation from the circulation to distant sites, and 5) adaptation to and proliferation in a new environment [2–4]. Due to the complexity of this process, metastasis is a highly inefficient process [5, 6]. During each step of the metastatic cascade, mutant and therefore potentially immunogenic cancer cells can be recognized and killed by the host immune system [7]. For example, antigens expressed by the primary tumor cells may be presented on MHC-I molecules and recognized by cytotoxic T cells (Box 1), leading to T cell activation and their killing of the tumor cells [7, 8]. Unfortunately for the patient, cancer cells exploit several mechanisms to evade destruction by the immune system, enabling them to proceed through the metastatic cascade. Additionally, under certain circumstances some immune cells and their mediators in fact favor metastatic disease and tumor growth [9–13].

Our immune system is capable of recognizing potentially harmful pathogens by the means of antigens. The immune system is educated in such a way that it does not respond to our own antigens [14]. However, as cancer cells acquire a high number of mutations and alterations [15] they express tumor-specific antigens that can be recognized as non-self and thereby activate the immune system, eventually leading to the killing of cancer cells. Besides a direct effect on antigen alteration, mutations can alter protein quantity, processivity and subsequent antigen presentation, thereby favoring recognition by the immune system. In this way, the immune system is able to prevent the occurrence of primary tumors (through immune surveillance) and also the rise of metastasis (through mutation-specific immunity induced by the primary tumor). Over a century ago, murine models of metastasis showed that progressive growth of a primary tumor suppressed the growth of a newly implanted, secondary tumor through a mechanism involving the immune system, a phenomenon now known as concomitant immunity (CI) [16–19]. These data indicate the tumor can induce both an anti-tumor immune response, as well as immunosuppressive mechanisms (e.g. regulatory T cells (Tregs) and immune-suppressive stroma) that allow it to evade an attack by the immune system. However, any secondary metastatic tumors do not initially have the benefit of an immune-suppressive stroma and may not have developed the same defensive mechanisms as the primary tumor and are therefore more vulnerable to be detected and killed by the immune response. Interestingly, in some cases once the primary tumor was surgically removed, the inhibitory influence on metastatic growth was lost, indicating the primary tumor itself might also have a systemic inhibitory effect on metastasis.

Over the years, several hypotheses for the disappearance of CI after primary tumor removal have been proposed, including an increased activity of suppressor cells [20], and the secretion of inhibitory factors by the primary tumor suppressing the growth of metastatic cells [21–24]. In contrast, other cases showed that the removal of the primary tumor rendered mice immune to a subsequent graft of the same tumor cell line [20], indicating the primary tumor can induce persistent immunity to a secondary tumor. Interestingly, CI was found to not always be tumor specific [24, 25], indicating that besides T cells other CI mechanisms are in place to prevent metastasis. If so, those mechanisms would be highly clinically relevant as they would enable a broadly applicable approach to prevent metastasis.

As metastases are considered to be secondary tumors derived from the primary tumor after its establishment, concomitant immunity may be involved in controlling the occurrence of metastasis. Due to the fact that the immune system can both promote and inhibit metastasis, it is of great importance for the clinic to understand which mediators are involved and how they impart their effects, in order to identify new targets to prevent metastatic disease.

### Immune cells at the primary tumor site influence metastatic behavior of cancer cells

The infiltration of immune cells into the primary tumor can have positive or negative effects on the patient’s prognosis [26]. Tumors not only actively escape from the immune system, they can also co-opt certain immune processes. A major mediator of this co-opting process by the tumor is through modification of the tumor stroma. The stroma consists of several cell types that contribute to tissue homeostasis, including fibroblasts, endothelial cells, nerve cells, immune cells and the extracellular matrix (ECM). Normally, it provides tissue homeostasis by controlling the balance between cell proliferation and cell death through interactions with the extracellular matrix (ECM) and fibroblasts [27]. However in cancer, fibroblasts often induce tumor progression by stimulating the proliferation and invasive phenotype of cancer cells, increasing their metastatic potential [28]. In pancreatic cancer, the dense fibrosis (desmoplasia) has been postulated to play either an inhibitory role constraining tumor growth or a protective role by providing survival signals and possibly impeding drug delivery to the cancer cells [29–31]. Tumor stroma can also promote the formation of new blood vessels, a process called angiogenesis. Without angiogenesis, a solid tumor will be limited in size and in its ability to access the blood stream for dissemination, an essential aspect to metastasis. Angiogenesis is initiated when the balance between pro-angiogenic factors and anti-angiogenic factors changes in favor of the former; this is also known as the angiogenic switch.

Another principal cell type in the tumor stroma is the macrophage. In breast cancer, the density of tumor-infiltrating macrophages positively correlates with angiogenesis and poor prognostic outcome [32]. Experimental inhibition of macrophage infiltration into the primary tumor delayed the angiogenic switch, which could be restored by the genetic restoration of the infiltrating macrophage population through transgenic overexpression of macrophage-colony-stimulating factor (CSF-1) [33]. There are different types of tumor-associated-macrophages (TAMs), with pro- or anti-tumor activity (Box 2) [34]. TAMS can be recruited into the primary tumor by cancer cell derived chemokines and cytokines (e.g. CSF1, VEGFA, CXCL2, CXCL12). TAM1 macrophages are inflammatory and generally thought to be tumor suppressive. Conversely, TAM2 macrophages can decrease CD8^+^ T cell infiltration and are typically pro-tumorigenic [35]. A similar effect can be mediated by transforming growth factor (TGF)-β polarized tumor-associated-neutrophils (TANs) [36]. Both TAMs and TANs are thought to promote migration and intravasation of cancer cells [37, 38]. For example, IL-4-expressing CD4^+^ T lymphocytes indirectly promoted invasion and metastasis of mammary carcinoma by activating epidermal growth factor signaling in mammary adenocarcinoma cells and changing the phenotype of tumor-associated macrophages from TAM1 to TAM2 [12]. On the other hand, macrophages that were activated as a consequence of T-cell-mediated immunity systemically inhibited the growth of both related and unrelated secondary tumors [39]. These experiments indicate that shifting the balance from pro-tumor TAMs and TANs to their anti-tumor counterparts can prevent metastasis and may have clinical potential.

–Beyond macrophages, other immunosuppressive cells in the tumor stroma enable metastasis by limiting immunosurveillance at the primary tumor site. An important example is the immunosuppressive CD4+ CD25+ Treg (Box 3). Tregs limit immune responses to normal tissues, thereby preventing auto-immunity, but this immunosuppressive function is frequently co-opted by tumors to inhibit immune destruction and promote metastasis. In some instances, the recruitment of Tregs to the primary tumor is necessary for metastasis [40, 41]. By producing immunosuppressive cytokines such as TGF-β and IL-10, the tumor can favor Treg proliferation and survival over anti-tumor T cell subsets within the tumor microenvironment [42]. Subsequently, Tregs inhibit the differentiation and proliferation of cancer killing (cytotoxic) effector CD8+ T cells through inhibition of IL-2 production [43] and inhibit the maturation and antigen presenting function of dendritic cells (DCs) [44]. Tregs directly inhibit CD8+ T cell-mediated cytolysis through TGF-β dependent inhibition of degranulation [45]. Additionally, under circumstances of strong CD8+ T cell priming, e.g. in cancer-vaccine settings, Tregs by regulating IL-2 homeostasis, limit CD8+ T cell responsiveness to IL-2 thereby preventing their expansion and survival [43].

The presence of Tregs can directly suppress CI in experimental models. Mice bearing poorly immunogenic B16 melanoma are not protected from a second tumor challenge, suggesting lack of CI. However, depletion of Tregs was sufficient to uncover CD8+ T cell-mediated CI against a secondary inoculated B16 tumor [46]. These data were confirmed by inducing B16 tumors in RAG1^−/−^ mice (lacking mature B and T cells) infused with CD8^+^ and CD4^+^ T cells lacking the CD4^+^ CD25^+^ Treg compartment, which induced robust CI, and which could be suppressed by the re-addition of CD4^+^ CD25^+^ Treg cells. These results suggest that concomitant tumor immunity can prevent growth of secondary tumors, even if they are only weakly immunogenic, as long as Treg activity is inhibited [46].

Besides their effect on CD8^+^ T cells, Tregs can directly inhibit natural killer (NK) (Box 1) cell effector functions through membrane-bound TGF-β and consequent down-regulation of NKG2D receptors on the surface of NK cells, without which NK cells do not efficiently recognize tumor cells [47, 48]. Chemokine receptor 4 (CCR4) positive Tregs are also able to induce NK cell apoptosis by secretion of the β-galactoside-binding protein (LGALS1), an anti-proliferative cytokine [49]. Results emphasizing this interaction between Tregs and NK cells are found in experiments showing that the depletion of Tregs, as seen with metronomic cyclophosphamide treatment, leads to an increase of NK cells. Thus, Tregs are able to counteract cancer-killing immune cells from both the adaptive and innate immune systems, and as a consequence inhibition of Tregs may prevent metastasis.

### Interactions between disseminated cancer cells and specific immune cells in the circulation

One plausible explanation for the occurrence of CI is that the primary tumor has a well-established immune-suppressive environment consisting of Tregs and macrophages in the tumor stroma, while disseminating or freshly implanted cancer cells do not initially possess a local immune-suppressive environment. This would explain why secondary tumors do not arise, as they are attacked and killed by the immune system before they can establish a local immunosuppressive microenvironment. Several distinct immune cell subsets can kill tumor cells in the circulation, and tumor cells accordingly employ specific mechanisms to survive.

### T cell-mediated concomitant immunity

To form metastases, the migrated and intravasated cancer cells need to reach distant sites while surviving stressful conditions such as shear forces and anoikis and attacks by immune cells in the blood stream. While thousands of cancer cells can reach the circulation every day, only a very small percentage will survive and have the capacity to form metastases [50, 51]. Early experiments identified an antitumor CD8^+^ T cell response against early disseminated mastocytoma tumor cells that delayed metastatic onset compared to tumors growing in T cell deficient mice [52]. While the CI-response initially decreased the number of metastatic cells in the lymph nodes and spleen by 90%, the number of metastatic cells subsequently increased as the CI-response dwindled. These results emphasized the importance of CI as a defense mechanism against metastasis. A more recent study confirmed such findings in an in vivo spontaneous metastatic melanoma model. Tumor cells disseminated early, and adopted a dormant, senescent state allowing them to survive in distant tissues without proliferating. Upon depletion of cytotoxic CD8^+^ T cells metastatic growth increased, indicating a role of the immune system in inhibiting tumor cell proliferation after dissemination [53]. Nonetheless, both studies did not elucidate why the CI-response declined over time and whether tumor cells were actively escaping CI by gaining properties of immune escape. This raises a question whether resistance against CI exists and how this is mediated in the circulation. The answers may point to new therapeutic targets for preventing metastatic disease.

### Mechanisms of defense against cytotoxic T cells and NK cells: Recognition, function, adhesion

One mechanism by which disseminated cancer cells can render themselves invisible from T cells is through down-regulation of MHC Class I molecules, without which CD8+ T cells cannot recognize them [54]. Down-regulation of interferon regulatory factor 7 (Irf7) in breast cancer cells further decreases MHC molecule expression on tumor cells further enhancing immune escape and promoting bone metastasis [55]. In mice lacking IFN-receptor or CD8^+^ T cells and NK cells, metastasis was accelerated confirming that Irf7 suppresses metastasis through IFN.

Another way tumors can avoid their destruction in the circulation is by preventing their binding to circulating immune cells. NK cells recognize reduced MHC Class I expression as a sign of “missing self”, triggering them to attack these cells through the release of cytotoxic granules [56]. However, tumor cells can limit NK cell mediated tumor cell death through reduced expression of adhesion proteins required for productive tumor-immune cell interaction. For example, expression of ICAM-1 or ICAM-2 by cancer cells is necessary for leukocyte adhesion and subsequent killing [57, 58]. Thus, in neuroblastoma, ICAM-2 expression confers a non-metastatic phenotype [59] [60]. Potentially, loss of ICAM-2 expression in disseminated tumor cells allows their evasion of the immune system, allowing metastases. Indeed, treatment of a peritoneal metastasis model of gastric cancer with adenovirus expressing ICAM-2 reduced the number of metastatic nodules [58].

Another example is the NKG2D receptor, an activating receptor found on NK cells (and also on CD8 T cells, NKT cells and subsets of γδ T cells). NKG2D ligands are expressed by cells in stress, including infected or tumor cells. Binding of a NKG2D ligand activates NK cells and results in death of the stressed cell. Data from mouse models supports this classical understanding of NKG2D function. In xenograft models of cancer cell lines, expression of NKG2D ligands resulted in tumor rejection [61, 62] and an antibody blocking NKG2D increased the growth of methylcholanthrene (MCA)-induced fibrosarcoma [63]. Yet there has been contradicting clinical data in cancer of the immuno-suppressive role of NKG2D. Many cancers express NKG2D ligands and yet still progress, suggesting they are not sufficient to mediate tumor regression. Multiple ligands of NKG2D have been shown to correlate with improved patient survival in colorectal and early stage breast cancer [64, 65], yet in high-grade invasive breast [66] and ovarian cancer [67], other NKG2D ligands have been shown to correlate with poor prognosis. It has been suggested that the difference in response is due to different actions of the membrane-bound and soluble forms of the ligands of NKG2D. Liu et al. demonstrated this in a humanized mouse model, exploiting the ability of the human NKG2D ligand MICB to activate mouse NKGD2 [68]. They developed two models, one expressing the native form of MICB that can be shed, and a mutated form unable to be shed from the membrane. The membrane-restricted MICB provided protective immunity and prevented spontaneous tumorigenesis, while the shed/soluble form facilitated tumor progression. However, since this study was published, Deng et al. demonstrated that a shed NKG2D ligand was capable of promoting NK cell activation and tumor rejection [69]. This may be a result of the different identity of the ligands studied, MULT1 (found in mice only) compared to the human ligand MICB, or hint at an added layer of complexity still to be understood. While utilizing the anti-tumor immunity of NK cells through NKG2D initially appeared attractive, a better understanding will be necessary of the difference responses to the membrane bound and soluble forms of the ligands, and of the different responses different ligands induce.

Alternatively, disseminated cancer cells can make use of the coagulation response to shield themselves from immune attack [70]. Studies of metastasis formation in mice lacking the Gαq protein critical for platelet activation, uncovered a correlation between platelet function and metastasis. Platelet function increased the survival of circulating tumor cells by impeding NK cells, as the depletion of NK cells in control mice harbored a phenotype comparable to the Gαq-deficient mice [71]. However, the study did not elucidate the mechanism by which platelet activation impedes NK cell function, hypothesizing it creates a physical barrier between circulating cancer cells and NK cells as direct contact is needed to enable NK-mediated cell lysis. NK cells express receptors capable of binding to platelet-derived factors such as PDGF, leaving open a role for these factors to directly inhibit NK cell function in the circulation [72]. Interestingly, another regulator of coagulation, tissue factor (TF), was found to play a role on several levels in the metastatic cascade. Not only is TF thought to favor angiogenesis [73], it could also play a promoting role in tumor cell migration [74] and survival of circulating cancer cells through increasing the aforementioned platelet-directed hindrance of NK cells [75]. The knock-down of TF in osteosarcoma cell lines resulted in a decrease in IL-8 and CXCL1 expression [74], both involved in neutrophil recruitment which could help to promote metastasis through suppression of the effector functions of cytotoxic CD8^+^ T cells [76]. These data indicate that coagulation factors in the circulation may link metastasis and the immune system, and can be used by cancer cells to evade CI in the circulation.

It is thought that CI includes at least two different mechanisms of inhibiting metastasis: one is induced by small immunogenic tumors and consists of a tumor-specific CD8^+^ T cell response, and the other is induced by larger immunogenic or non-immunogenic tumors and consists of non-specific serum-mediated mechanisms [77, 78]. Both mechanisms can be counteracted by cancer cells to evade CI and enable metastatic growth. For example, primary breast cancer tumors increase their own ability to metastasize by inducing systemic inflammation through IL-1β, which induces the expression of IL-17 from γδ T cells, leading to the expansion and polarization of neutrophils through granulocyte colony-stimulating factor (G-CSF) dependent mechanisms. These tumor-induced neutrophils are able to systemically suppress the effector functions of cytotoxic CD8^+^ T cells, thereby promoting metastasis [76]. The neutralization of IL-17 or G-CSF and the absence of γδ T cells or neutrophils reduced metastasis from occurring. This is an example of the influence the primary tumor can have on survival of disseminated metastatic cells in the circulation and may be one of the mechanisms tumors use to circumvent CI. Importantly, the elucidation of the molecular mechanism allows for therapeutic targeting of metastasis, since approved inhibitors of IL-1 and IL-17 are available for clinical use.

Tregs not only promote metastasis through inhibition of cytotoxic CD8^+^ T cells and NK cells in the primary tumor, but also block the function of circulating CD8^+^ and NK cells against circulating metastatic cancer cells [79]. However, while much research has focused on the impact of infiltrating Tregs on cancer progression, there are few reports on the effect of circulating Tregs on metastasis and clinical prognosis. This is surprising given the major role of Tregs in cancer progression of the primary tumor. One report showed an increase in circulating Tregs after treating metastatic renal cell carcinoma patients with low dose IL-2 in a dendritic cell vaccination setting [80], but whether or not these Tregs affected tumor progression was not addressed. Another study evaluated the pre-treatment frequency of Tregs, and showed no correlation with clinical response to anti-cancer vaccination with PROSTAVAC, a virus based vaccine that carries prostate specific tumor-associated antigen PSA, in prostate cancer patients [81]. The effect of circulating Tregs on metastasis and tumor progression should be further investigated, as the reduction of Tregs in primary tumors is an intensely pursued therapeutic goal. Interventions to limit Treg infiltration into established tumors should be balanced with the potential for accumulation of Tregs in the circulation and in normal tissues, where they might suppress CI and thereby promote the survival and implantation of circulating tumor cells.

A serum-based mediator of CI induced by immunogenic and non-immunogenic large tumors is tyrosine isomer serum factor, consisting of meta-tyrosine and ortho-tyrosine derivatives of the much more abundant common amino acid, (para-)tyrosine. It is thought tyrosine isomers are produced by the primary tumor, and inhibit the proliferation of disseminated cancer cells through inhibition of the MAP/ERK pathway and inactivation of STAT3. This potentially drives cancer cells into a state of dormancy in G(0)-phase, thereby allowing more nutrients to favor the high metabolic rate of the primary tumor. Other possible mechanisms would involve the activation of an S-phase checkpoint, also inhibiting disseminated cancer cell proliferation by accumulating cells in S-phase [82]. Inhibition of STAT3 activity also abrogates multiple mechanisms of immune suppression, possibly linking the direct effect of tyrosine isomers on the cancer cells with their activity against immune suppression. Tyrosine isomers could be tested as therapeutics in a setting of surgical resection of the primary tumor to suppress outgrowth of existing micrometastases. In summary, understanding the multiple mechanisms of resistance to CI in the circulation may point to interventions that block the systemic spread of metastatic cells through the circulation.

### Formation of the metastatic niche and the role of immune cells

For metastases to grow out, the circulating metastatic cells need to exit the circulation by extravasation, and adapt to their new environment. Interestingly, many cancer types preferentially metastasize to defined secondary locations, indicating the spread of metastases is not random [83]. Important mediators of this selective metastatic localization of cancer cells are chemokines, secreted proteins which also control the trafficking of leukocytes [84]. Through interaction with G-protein-coupled receptors, chemokines induce cytoskeletal rearrangement, integrin adhesion, and directional migration [84], all of which are important for homing of metastatic cancer cells to distant sites. Several investigations report a role for the chemokine receptor CXCR4 and its ligand CXCL12 in site-specific metastasis [84–87], where neutralization of the CXCL12/CXCR4 interaction significantly impaired metastases formation in lymph nodes, bone, and lung in metastatic breast cancer models [84, 87]. While CXCR4 is expressed in many cancers including breast cancer, melanoma, and colorectal cancer [84–86, 88], little is known about the regulation of its ligand, CXCL12. Currently, the chemokine receptor-ligand axis seems to serve an important role in localizing the metastasis, as chemokines produced in specific organs increase the adhesive, invasive, and migratory properties of circulating tumor cells expressing the chemokine receptor. The chemokine receptor-ligand axis also plays an important role for immune cell trafficking. For example, CXCR4 plays a central role in trafficking of Tregs [89]. This further emphasizes the importance of the chemokine receptor-ligand axis in localizing metastasis, as it could induce a pro-tumor immune environment. Thus, the therapeutic potential of chemokine inhibition to prevent cancer cell metastasis strongly depends on the simultaneous effects on immune cells. A better understanding of the regulation of pro-metastatic chemokine expression in target organs, and its effect on trafficking of both tumor and immune cells, will allow rational therapeutic intervention to prevent metastasis.

Another requirement for metastatic growth is survival of metastatic cancer cells in their new environment. Before cancer cells are able to engraft in a secondary tissue, the environment of the target tissue needs to change in order to create a permissive microenvironment; the metastatic niche (the seed and soil hypothesis; metastatic cells, (seed), typically prefer a specific tissue (soil), for engraftment) [90]. The pre-metastatic niche can be prepared by the primary tumor through tumor conditioning of bone-marrow derived myeloid cells in the target tissue [91, 92]. Not only do bone-marrow derived myeloid cells infiltrate the primary tumor to promote metastasis, they also accumulate at distant sites marking the metastatic niche to promote adhesion through VEGFR1 mediated clustering, and tissue invasion through matrix breakdown by matrix metallopeptidase 9 (MMP9), thereby promoting metastatic growth [91, 93]. In a model for metastatic breast cancer, tumor-specific CD4^+^ T cells create a metastatic niche in bone by inducing osteolytic bone disease and subsequent release of growth factors through RANKL mediated mechanisms [94]. When inhibiting RANKL-secreting tumor-specific CD4^+^ T cells, bone metastases but not metastasis to other organs was decreased, indicating a site-specific mechanism. In another preclinical mouse model for metastatic breast cancer, the complement anaphylatoxin C5a receptor (C5aR) on immune cells facilitated metastasis to the lungs by suppressing local CD4^+^ and CD8^+^ T cell anti-tumor responses through recruitment of immature macrophages to the metastatic niche. By secreting TGF-β and IL-10, these macrophages favored the differentiation of Tregs from the CD4^+^ T cell subset, leading to the inhibition of Th1 cells and CD8^+^ T cells. In C5aR-deficient mice, the local T cell response was sufficient to reduce lung metastasis, and the depletion of CD8^+^ T cells reversed this beneficial effect [94, 95]. The combination of C5aR deficiency and depletion of tissue resident macrophages synergized, leading to increased protection against lung metastases [96]. These studies indicate that tissue resident macrophages are an important aspect of the metastatic niche by inducing local immunosuppression [87] and thereby help circumvent CI.

Beyond T cells and macrophages, NK cells also play a prominent role in the metastatic niche. In a B16 metastatic murine melanoma model, different subsets of NK cells were found to influence the occurrence of metastasis to certain sites, as NK cell depletion increased metastasis to the liver without affecting metastasis to the lung [97]. A significant difference was found in the distribution of NK cell subsets, as defined by their expression of CD27 and CD11b, in the lung and liver. The CD27^+^ CD11b^−^ immature NK subset in the liver was found to protect against liver metastasis, but not pulmonary metastasis, through a NK cell perforin-dependent cytotoxic mechanism, while the (CD27^−^ CD11b^+^) mature NK cell subset found in the lung whilst unable to efficiently prevent metastasis formation, yet controlled tumor load (fewer pulmonary nodules). These data indicate that organ-specific immune responses determine the permissiveness of a certain metastatic niche [97]. Other investigations show the inhibition of NK cells is needed to form a metastatic niche, and is induced by hypoxic conditions in primary tumor cells. This leads to the secretion of pro-angiogenic factors and cytokines, which direct CD11b^+^ Ly6C^med^ Ly6G^+^ myeloid cells to the metastatic niche where they inhibit NK cell maturation and impair their cytotoxic capacity [98, 99].

Due to the site-specific involvement of immune cells in this last step of metastasis, it may prove challenging to intervene with therapeutically. Opportunities lie in combination therapies acting on several immune players needed for the attraction of metastatic cells in all of the different metastatic niches. Directions include neutralization of the CXCL12/CXCR4 axis, inhibiting VEGFR1-positive myeloid cells, or promoting specific NK subsets in specific organs, for example with cytokines such as IL-15. It will be interesting to learn whether the known anti-metastatic activity of certain immune-based therapies (i.e. IFN-α therapy in stage 3 melanoma patients after surgery) or even conventional therapies (chemotherapy after breast cancer surgery) are in fact at least partially mediated by reconditioning the metastatic niche to make it less hospitable for newly arriving, circulating cancer cells [100, 101].

### Concomitant immunity as a therapeutic target to prevent metastasis

Concomitant immunity is the phenomenon of secondary tumor rejection during primary tumor growth, observed in many animal models of cancer. As we have outlined, CI can be induced by multiple tumor-derived/induced stimuli, and different immune cell subsets can either promote or inhibit metastasis. Important players are the T cells, NK cells, and M1-like macrophages which can recognize and kill metastatic cancer cells, and the Tregs and M2-like macrophages which are programmed by the tumor to circumvent CI through inhibition of T cells and NK cells. Multiple studies demonstrate how inhibition of specific CI mechanisms accelerates metastatic growth. Therefore, increased understanding of CI may provide several new targets for cancer therapy.

Concomitant immunity appears to frequently weaken as time progresses, and metastasis occurs [18]. For instance, one study demonstrated that macrophages isolated at different time-points in the course of CI have different effects on artificial mammary carcinoma lung-metastases formation. When administrating macrophages from the late period of CI, the anti-metastatic effect seen with early macrophages was lost either due to loss of their cytotoxic activity or by a shift from cytotoxic to immunosuppressive macrophages. Inhibition of prostaglandin E2 synthesis restored the anti-metastatic effect of the late CI macrophages [18]. This example highlights the importance of mechanistic studies, as they directly suggest specific interventions to bolster CI against metastasis. For example, specific inhibition or depletion of Tregs would strengthen cytotoxic CD8^+^ T cell and NK cell function and/or numbers in both the primary tumor and in the circulation. This could prevent the initial dissemination of cancer cells from the primary tumor, while also increasing the anti-tumor effect against already disseminated tumor cells in the circulation or newly seeded cancer cells at distant sites. The specific inhibition of Tregs has yet been unsuccessful, as many interventions also negatively affect other anti-tumor immune cells. Interestingly, recent evidence suggests that isoform-specific inhibition of the PI3K-Akt pathway preferentially inhibits Tregs with minimal effect on conventional T cells both in vitro and in vivo [102], resulting in increased anti-tumor activity. Controlling Treg trafficking may also be an interesting but as of yet under-investigated way to reduce the immunosuppressive effects caused by the primary tumor. When the Tregs are redirected to the circulation, CD8^+^ T cells and NK cells may be unleashed in the primary tumor to prevent the release of cancer cells into the circulation, thereby preventing metastasis. Some studies hypothesize that the blockade of CXCR4 might lead to a block in Treg trafficking. One group has shown that in human ovarian cancer, tumor-associated microphages produce chemokine CCL22, which mediates Treg cell trafficking. Blockade of CCL22 in vivo significantly reduced human Treg migration in ovarian carcinoma [89]. Nonetheless, as cancer cells disseminate early in cancer progression, the risk of this approach would be that already circulating metastatic cancer cells would be protected by circulating Tregs and form metastases more readily.

Since many chemotherapeutics kill highly proliferative cells, chemotherapy could shift the balance from Tregs to effector T cells as a higher frequency of proliferating cells is observed within the Treg versus the non-Treg populations of CD4^+^ T cells [103]. A more recent study shows that the chemotherapeutic drug cyclophosphamide induces the expression of CXCL3 by tumor cells, leading to intratumoral migration of CD4^+^ T cells expressing cytotoxic molecules, which are able to eradicate the tumor through specific tumor immunity [104]. Thus, chemotherapy may have positive effects on tumor-specific immunity. However, as chemotherapy can also kill beneficial immune cells such as CD8^+^ T cells, more research is needed to investigate specific mechanisms and optimal dosing and scheduling for individual chemotherapeutics. Another interesting combination therapy, combining ionizing radiation and CTLA-4 blockade, demonstrated an immune-mediated inhibition of metastasis by favoring the induction of CD8^+^ T cells over CD4^+^ T cells [105]. Ionizing radiation kills tumor cells, causing the release of tumor specific antigens, leading to priming of tumor-specific CD8^+^ T cells that kill more tumor cells [106, 107]. In addition, CTLA-4 is expressed on both regulatory and activated T cells, and by blocking of CTLA-4 on both CD8^+^ T cells and Tregs, a synergistic effect can lead to maximal anti-tumor activity, through enhancement of CD8^+^ T cell effector function together with inhibition of Treg function [108]. Finally, anti-CTLA-4 mAbs can bind to highly expressed CTLA-4 on intratumoral Tregs, causing their killing through ADCC by macrophages [109]. This illustrates how conventional therapies can be used, alone or in combination with immunotherapies, to target Tregs. While many of these combination therapies are intensely studied in preclinical and clinical scenarios, the readouts are often the anti-tumor immune response and its effect on the primary tumor. Metastasis is much less studied, and it will be important to learn whether and how these strategies impact metastasis, since it is typically the ultimate cause of mortality in most cancers.

NK cells also play distinct roles in CI and could therefore be interesting targets for therapy. A recent study showed a fascinating role effect of BRAF inhibitors on NK cells in preventing metastatic melanoma. Resistance of cancer cells to BRAF inhibitors limits their therapeutic efficacy, and immune-based therapies might help overcome relapse. The anti-metastatic effects of the BRAF inhibitor PLX4720 required host natural killer (NK) cells and perforin in vitro, where PLX4720 enabled NK cell proliferation. Additionally, PLX4720 treatment significantly enhanced NK cell frequencies in BRAF (V600E) lung metastases [99], suggesting that additional NK cell-based therapy might elicit more durable responses to BRAF inhibition. However, as previous combination therapies (BRAF inhibitor with immune-checkpoint-inhibitor PD-1) exhibited high toxicity [110], it is extremely important to understand the interactions of different drugs. These data again indicate the importance of going beyond inhibiting or enhancing a certain immune cell subset with a single therapeutic with a focus on the primary, established tumor(s), to include studies on the effects of combination therapies against metastasis.

Other ways to potentially improve CI in order to prevent metastasis, would involve targeting TAMs and TANs. As previously mentioned, TAMs and TANs can promote the migration and intravasation of cancer cells in the primary tumor [37, 38] while also decreasing CD8^+^ T cell infiltration [35, 36]. Additionally, macrophages play a role in the formation of the metastatic niche by locally suppressing the immune system [96]. Therefore, suppression of pro-tumor macrophages could benefit CI by interfering with every step of the metastatic cascade. A recent study using resveratrol, a compound that indirectly inhibits pro-tumor macrophage (M2) activation, indicated it had anti-metastatic effects [111]. Similarly, the selective TAM inhibitor CNI-1493, which inhibits the production of macrophage-derived inflammatory mediators, also demonstrated an anti-metastatic effect through the inhibition of cancer cell extravasation [112]. Thus, the inhibition of macrophages could have clinical anti-metastatic potential. However, the coexistence of pro-tumor (M2) and anti-tumor (M1) macrophages in tumors demands commensurate specificity of therapeutics that target them to inhibit pro-tumor macrophages and bolster their anti-tumor counterparts.

Beyond immune cells, the coagulation system is an anti-metastatic target given its role in shielding the disseminated cancer cells from immune cells in the circulation [70] [75]. A recent review concluded that clinical evidence concurs with experimental evidence that inhibition of platelets leads to a decrease in metastasis, suggesting that the coagulation system might harbor several targets for new therapies, such as TF and PDGF [113]. Since inhibition of coagulation may function through “unshielding” tumor cells for attack by immune cells, this may be especially powerful in the context of therapies that activate these immune cells.

Taken together, CI suppresses multiple steps in the process of metastasis, a finding that points to possible clinical interventions (overview given in Table 1). However, each therapeutic intervention will require careful investigation of the effects on individual immune cell subsets, ensuring that pro-metastatic immune cells are inhibited, while not affecting, or ideally promoting, the activity of their anti-metastatic counterparts.

**Table 1:** Overview of immune-cell subsets and clinical applications in preventing metastasis

Cell Subset	Primary tumor: Pro- metastatic	Primary tumor: Anti-metastatic	In the circulation: Pro- metastatic	In the circulation: Anti- metastatic	Metastatic niche: Pro- metastatic	Metastatic niche: Anti- metastatic	Clinical applications
CD8^+^ T cells	–	Recognize and kill cancer cells prior to dissemination.	–	Recognize and kill disseminated cancer cells.	–	Recognize and kill cancer cells upon entry into the metastatic niche.	Stimulation of CD8^+^ T cells: - Vaccination - Engineering the T cell receptor (TCR) or chimeric antigen receptor (CAR); while this is a promising way to target the primary tumor, it remains to be seen to what extent they also control metastatic cells, which may have altered antigenic properties.
Tregs	Immuno-suppression through TGF-β and IL-10 secretion, inhibiting cytotoxic T cell and NK cell responses.	–	Could deplete or suppress circulating T cells and NK cells to improve survival of circulating metastatic cells. [133, 134]	–	–	–	Inhibition of Tregs: - PI3K-Akt inhibitors [102] - FOXP3 inhibitors [103] Treg inhibition in mice can lead to autoimmune disease, necessitating caution or targeted, local Treg depletion (as opposed to systemic depletion)
NK cells	–	Recognize and kill cancer cells prior to dissemination.	–	Recognize and kill disseminated cancer cells.	Mature NK cells have fewer killing capacity. Therefore, niches with mature or exhausted NK cells are more prone to promote metastatic outgrowth.	Immature NK cells (CD27^+^ CD11b^−^) prevent metastatic niche formation.	Stimulation of NK cells: Adoptive transfer [120] or cytokines such as IL-2, IL-15, IL-21 [135] Clinical benefit, and the advantage over T cells that no priming is needed. Combinations of stimulating NK cell function and blocking NK cell inhibition by Tregs or platelet-mediated shedding could improve NK cell-mediated prevention of metastasis.
Macrophages	Angiogenesis - Immuno-suppression - Promote tumor cell migration and intravasation	Direct tumor cell killing, antigen presentation to T cells.	Shedding of metastatic tumor cells from the immune system.	Immuno-stimulation: produce IL-12, IL-6 and CXCL9 to stimulate the immune system. Express iNOS to kill tumor cells directly through production of nitric oxide.	Site-specific metastasis by preparing the metastic niche through induction of local immune-suppressive environment.	Local immune-stimulation	Inhibition of macrophages: - CSF-1 inhibition to inhibit infiltration in the primary tumor. - Influence polarization to favor M1 over M2, e.g. with TLR agonists [121] Caution must be taken to selectively inhibit M2 macrophages while preserving or enhancing M1 macrophage activity.

## Conclusion and perspectives

Immunotherapy has gained a prominent place in the therapy of multiple cancers due to the initial successes of antibody blockade of CTLA-4 (with Ipilimumab) and anti-PD-(L)1 (with Nivolumab, Pembrolizumab and Atezolizumab) in patients with metastatic cancers [14, 110, 111, 114]. In large part, these therapeutics appear to increase the already existing, spontaneous anti-tumor immune response against the primary tumor and (micro)metastases, long known as CI. The most prominent players in CI are cytotoxic CD8^+^ T cells, NK cells, and M1-like macrophages actively inhibiting metastases by recognizing and killing disseminated cancer cells in the early metastatic phase at the primary tumor as well as during later metastatic stages in the circulation. On the other hand, Tregs and M2-like macrophages can inhibit CD8^+^ T cells and NK cells, promoting metastases. Not only the primary tumor but also the plastic nature of individual immune cells and functions can shift the tumor immune microenvironment towards an immunosuppressive, pro-tumor environment, weakening CI and enabling immune escape. This suggests specific therapeutic approaches to influence this shift, either by inhibiting immunosuppressive cytokines such as CSF1, CXCL12, TGF-β, or IL-10 produced by the primary tumor, specific inhibition of Tregs and M2-like TAMs, or by promoting the tumor-specific activity of M1 TAMs, CD8^+^ T cells and NK cells. As an example, genetically engineered T cells expressing T cell receptors (TCR) that recognize specific tumor antigens are tested in patients with metastatic cancer [115, 116]. While this is a promising way to target the primary tumor and macrometastases, it is also important to investigate the recognition of metastatic cells by such engineered T cells, as metastatic cells may have different properties in order to enable metastasis in the first place. For example, even if the primary tumor and/or macrometastases are not effectively treated with such an approach, it could still be effective in preventing new metastases, which would be especially valuable when detectable disease is limited or could be effectively controlled. One helpful measurement could be the impact of (immune) therapy on immune cell subsets as well as the number of circulating tumor cells, and correlating this with the subsequent development of metastases [117].

One of the biggest drawbacks of most preclinical models of CI is the use of transplanted secondary tumors to mimic metastases. While this approach is rapid and reproducible and enables the investigation of some critical aspects of tumor-specific CI responses, it incompletely models the patient situation, where metastases arise from single tumor cells. Specifically, the injection of thousand to millions of tumor cells to form a secondary tumor results in a massive release of antigens and accompanying immune-active signaling molecules from dying tumor cells, with unclear but likely profound effects on CI [118]. In addition, the naturally occurring metastatic processes of tumor cell detachment from the primary tumor, intravasation, survival in the circulation, and extravasation into the target tissue are all not recapitulated in models where direct injection of a secondary tumor cell inoculum simulates metastasis. Models of spontaneous metastasis, such as the classic 4 T1 breast cancer, or more recent genetically engineered mouse models typically take some time to develop true metastases arising from the primary tumor, but they allow the investigation of all the different steps of the metastatic cascade and the impact of CI throughout those steps [119]. Additionally, the immune system is found to play a role in most cancers, while preclinical CI research has classically been dominantly focused on melanoma and breast-cancer models. Another caveat is that the activation of the immune system can also promote metastasis if systemic inflammation is induced, possibly through activation of immune cells that prepare the metastatic niche [25, 109, 120, 121]. Therefore, combination therapies (e.g. suppressing Tregs while enhancing tumor-specific CD8^+^ T cells) require careful verification in multiple animal models before clinical application.

In conclusion, CI plays an important and diverse role in all steps of the metastatic cascade. Multiple specific targets in the interaction between CI and the metastatic cascade have been identified, allowing for the rational design of interventions that strengthen the anti-metastatic potential of CI to prevent cancer metastasis and thereby reduce cancer morbidity and mortality.

## Box 1 Cytotoxic T cells and Natural Killer cells in tumor recognition and killing

Immune-mediated tumor killing is found in the primary tumor [122] as well as in disseminated cancer cells (thereby contributing to concomitant immunity). Two important players in this direct immune-mediated tumor killing are CD8^+^ cytotoxic T cells (adaptive immune system) and natural killer cells (NK cells) (innate immune system).

For CD8^+^ cytotoxic T cells to be able to recognize and kill cancer cells, they first need to be activated and primed by recognition of tumor-derived antigens, presented by antigen presenting cells (APCs) such as dendritic cells (DCs). Normally, host proteins (self-antigens) are not well recognized by T cells due to normal processes of immune tolerance to self-antigens. However, cancer cells express mutated proteins (neoantigens) that can be recognized by T cells [123]. Once a CD8^+^ T cell recognizes the tumor-antigen-MHC-I-complex through its T cell receptor (TCR), in presence of the appropriate co-stimulation provided by the APC, T cell priming and activation will occur. This leads to CD8^+^ T cell proliferation, creating a cytotoxic effector T cell pool which is able to recognize all cells expressing the tumor-specific antigen, and kill them through the induction of apoptosis (through the perforin-granzyme B and/or Fas-Fas ligand axis) [124].

NK cells do not recognize tumor-specific antigens, and therefore do not need to be primed. Rather, NK cells directly recognize cancer cells through antigen-specific receptors such as NKG2D, NCRs, DNAM1 and CD16, which recognize ligands expressed on the cell surface, especially on stressed cells such as cancer cells. Additionally, NK cells recognize ‘missing-self’ which is induced by most tumors to evade T cell recognition by down-regulation of MHC molecules. Once a NK cell recognizes a cancer cell, it will induce apoptosis through granule-mediated-exocytosis or the Fas-Fas ligand axis, similar to cytotoxic CD8^+^ T cells [125]

## Box 2 Macrophages; whose side are they on?

Once monocytes exit the blood, they can become macrophages (M0). Under the influence of local cytokines such as IL-4, IL-6, IL-10, they can polarize and become M1 or M2 macrophages. Originally, it was thought that two types of tumor-associated macrophages (TAMs) existed; the anti-tumor M1 TAMs and the pro-tumor M2 TAMs [32, 126]. However, recent evidence suggests several distinct TAM populations exist, with properties of both M1 and M2 TAMs [127]. The anti-tumor M1 TAMs produce IL-12, IL-6 and CXCL9 to stimulate the immune system [128], and express iNOS to kill tumor cells directly through production of nitric oxide. M2 TAMs promote angiogenesis by producing IL-10 and CCL22, induce immune-suppression by inhibiting NK cells, T cells, and DCs by arginine deprivation through arginase expression, facilitate invasion by remodeling the stroma through matrix metalloproteases, and increase metastatic tumor cell shedding through abnormal tumor vasculature [12, 128], all of which are important factors for metastasis. Therefore, even though specific inhibition of M2 macrophages is challenging, it could be a very potent target to prevent metastasis.

## Box 3 Tregs; gate-keepers of the immune-response

Regulatory T cells (Tregs) are mostly CD4^+^ T cells that express the IL-2 receptor chain-α (CD25) and the transcription factor forkhead-box P3 (FOXP3) [129]. A normal and critical component of maintaining immune cell homeostasis and preventing autoimmunity [130, 131], they also inhibit beneficial anti-tumor immunity. Their suppressive effects are mediated by the secretion of IL-10 and TGF-β, inducing cell-cycle arrest or apoptosis in effector T cells and NK cells, and inhibiting the co-stimulation and maturation of DCs. Tregs can also compete for T cell growth factors such as IL-2, and use direct cell contact to inhibit immune cells through CTLA-4 molecules [132].

## Abbreviations

APC: Antigen presenting cell
C5aR: Complement component 5a receptor
CD: Cluster of differentiation
CI: Concomitant immunity
CSF: Colony-stimulating factor
CTLA: Cytotoxic T lymphocyte-associated molecule
CXCL: Chemokine (C-X-C motif) ligand
CXCR: C-X-C chemokine receptor
DC: Dendritic cell
FOXP3: Forkhead box P3
IL: Interleukin
MAP/ERK: Mitogen-activated protein/Extracellular signal-regulated kinase
MHC: Major histocompatibility complex
NK: Natural killer
PI3K-Akt: Phosphatidylinositol-4,5-bisphosphate 3-kinase- AKT8 virus oncogene cellular homolog
STAT: Signal transducer and activator of transcription
TAM: Tumor-infiltrating-macrophage
TAN: Tumor-infiltrating-neutrophil
TCR: T cell receptor
TGF-β: Transforming growth factor beta
Th1: Type 1 T helper
Treg: Regulatory T cell
VEGFA: Vascular endothelial growth factor A
